# Effects of pre- and postnatal probiotic and ω-3 fatty acid supplementation on cytokine and chemokine responses to allergens and TLR ligands during infancy

**DOI:** 10.1186/s13223-026-01036-y

**Published:** 2026-05-05

**Authors:** Cibely C Fontes-Oliveira, Amanda Nylén, Johanna Ljung, Astrid Welin, Lovisa Arvidsson, Magalí Martí, Dhanapal Govindaraj, Isabel García Martín, Camilla Janefjord, Lina Tingö, Ahmed Al-Kaabawi, Elisabet Severin, Karel Duchén, Maria C Jenmalm

**Affiliations:** 1 Division of Inflammation and Infection, Department of Biomedical and Clinical Sciences, Linköping University, Linköping, Sweden; 2 Allergy Center, University Hospital, Linköping, Sweden; 3 Division of Children’s and Women’s Health, Department of Biomedical and Clinical Sciences, Linköping University, Linköping, Sweden; 4 Present Address: Division of Cell and Neurobiology, Department of Biomedical and Clinical Sciences, Linköping University, Linköping, Sweden

**Keywords:** Allergy_1_, Lactobacilli_2_, Probiotic_3_, Infants_4_, Immunomodulation_5_, Cytokines_6_, Chemokines_7_

## Abstract

**Background:**

Reduced intensity and diversity of microbial stimulation and decreased intake of anti-inflammatory ω-3 polyunsaturated fatty acids (PUFAs) in Western diets may contribute to impaired postnatal immune development and increased allergy risk. Here, we hypothesize that early supplementation with probiotics and ω-3 PUFAs, starting during pregnancy and continuing during infancy, may promote appropriate immune maturation and thereby potentially prevent allergy development.

**Methods:**

In this study, 117 mother‒baby pairs were randomized into four groups receiving the following supplements: *Limosilactobacillus reuteri* (*L. reuteri*), ω-3 PUFA, double supplementation, or placebo. Supplementation started from gestational week 20 until 3 months of age (3 mo) for ω-3 PUFA and continued until 12 mo for *L. reuteri*. Peripheral blood mononuclear cells (PBMCs) from infants were isolated at birth and at 6, 12, and 24 mo, and stimulated *ex vivo* with several allergens and ligands of Toll-like receptors (TLRs). Cytokines and chemokines related to Th1/Th2/Th17/Treg responses were quantified.

**Results:**

Probiotic supplementation modulated the pattern of cytokine and chemokine secretion over time, whereas no clear effects were observed for ω-3 PUFA supplementation. *L. reuteri* supplementation led to a significant increase in Th1-associated C-X-C motif chemokine ligand 10 (CXCL10) levels induced by birch and cat allergens at 6 mo. Furthermore, *L. reuteri* induced more significant age-dependent changes under several types of stimulation than did the placebo, indicating enhanced immune maturation.

**Conclusion:**

Pre- and postnatal probiotic supplementation may promote immune maturation during early childhood.

**Supplementary Information:**

The online version contains supplementary material available at 10.1186/s13223-026-01036-y.

## Background

Allergic reactions are characterized by an uncontrolled immune response orchestrated by T helper (Th) 2 cells and triggered by innocuous environmental antigens [1]. Approximately 30% to 40% of the global population is affected by one or more allergic conditions, resulting in significant personal, social, and economic consequences [2, 3]. Allergies often begin early in life, and even during pregnancy, prenatal exposure to certain conditions may predispose the infant to the development of allergic diseases [4]. The onset of allergic diseases in childhood is typically progressive, with the appearance of the first symptoms during the first year of life, such as atopic dermatitis and/or food allergy. This phenomenon is known as the atopic march, and approximately 80% of individuals with asthma develop symptoms before the age of five [5]. The development of allergies throughout life can have severe consequences, potentially leading to chronic inflammatory conditions, impaired lung function, and disability in adulthood [2]. Multiple contributing factors are involved, including genetic predispositions, environmental influences, and the timing of allergen exposure [1, 6].

The composition of the gastrointestinal (GI) microbiota plays a crucial role in metabolism and immunological functions across tissues and organs [7]. Increasing evidence indicates that immune system maturation, a critical developmental process through which immune cells acquire appropriate functional competence, is profoundly influenced by early-life microbial colonization and maternal-derived factors such as antibodies and microbial metabolites [4, 8, 9]. These elements provide essential cues that guide the stepwise development of both innate and adaptive immune responses, shaping the immune system’s ability to distinguish between harmful and harmless stimuli. Proper immune maturation is essential for establishing immune homeostasis and long-term immunological resilience [9]. Conversely, disruption of microbiota maturation in early life may predispose individuals to the development of allergic diseases [10, 11].

The gut microbiota composition is strongly influenced by diet, and the increasing prevalence of conditions such as allergies and type 2 diabetes in affluent countries may be associated with a reduced dietary ω-3/ω-6 polyunsaturated fatty acid (PUFA) ratio [12]. A diet high in ω-6 fatty acids relative to ω-3 fatty acids may promote the production of proinflammatory mediators, such as prostaglandins and leukotrienes, and decrease the synthesis of immunoregulatory mediators [13].

Furthermore, the maternal microbiota ecosystem and microbial exposure during pregnancy are closely associated with the development and maturation of the infant immune system [4, 14]. Several studies, including those conducted by our group, have demonstrated the potential benefits of early-life microbiota modulation [15–17]. Notably, combined pre- and postnatal probiotic supplementation has shown promise in reducing the risk of allergy development [4, 18]. For example, we previously reported that the absence of *Lactobacillus* species in the infant gut microbiota is associated with increased allergy risk [19].

Previous clinical studies from our group have demonstrated that both ω-3 PUFAs and probiotics, such as *Limosilactobacillus reuteri* (*L. reuteri*), play important roles in modulating immune responses [15, 17, 19–22]. While ω-3 PUFAs exert significant anti-inflammatory effects, *L. reuteri* exhibits immunostimulatory properties that support immune maturation and help regulate allergic responses [23]. Notably, ω-3 PUFA supplementation during pregnancy significantly reduces the secretion of the proinflammatory mediator prostaglandin E2, particularly in nonatopic mothers, potentially lowering the risk of allergic inflammation in their offspring [20]. Building on these findings, further studies revealed that maternal ω-3 PUFA supplementation from gestational week 25 to 3 months *post-partum* significantly decreased the cumulative incidence and severity of Immunoglobulin E (IgE)-associated allergic diseases in infants up to two years of age [21]. These protective effects are associated with increased levels of docosahexaenoic acid (DHA) and eicosapentaenoic acid (EPA) in maternal and infant plasma, which are correlated with fewer and less severe allergic symptoms [21, 22]. Additionally, *L. reuteri* supplementation to the mother from gestational week 36 to delivery and then to the infant for the first 12 months has been shown to promote immune maturation and reduce IgE-associated eczema in infants [17]. Similar effects were also observed in studies using animal models of allergies supplemented with ω-3 PUFAs [24] and *L. reuteri* [25]. Collectively, these results suggest that a synergistic clinical approach combining ω-3 PUFAs and *L. reuteri* may offer a promising strategy for the prevention of allergic diseases in early childhood.

Even though pre- and postnatal probiotic and ω-3 PUFA supplementation have shown promising effects on allergy prevention, most studies have started supplementation in the third trimester [4, 17, 21]. Here, we hypothesized that a longer prenatal supplementation period, starting at week 20, may result in more effective allergy prevention through the modulation of immune maturation during infancy [4]. Therefore, the aim of the present study was to characterize the effects of supplementation with *L. reuteri* and/or ω-3 PUFAs, which are initiated at week 20 of pregnancy, on the development of cytokine and chemokine responses during infancy. These responses were assessed following targeted stimulation with allergens and Toll-like receptor (TLR) ligands to capture functional immune profiles relevant to early-life immune development and potential allergy-preventive effects.

## Methods

### PROOM-3 study

**Fig. 1:**
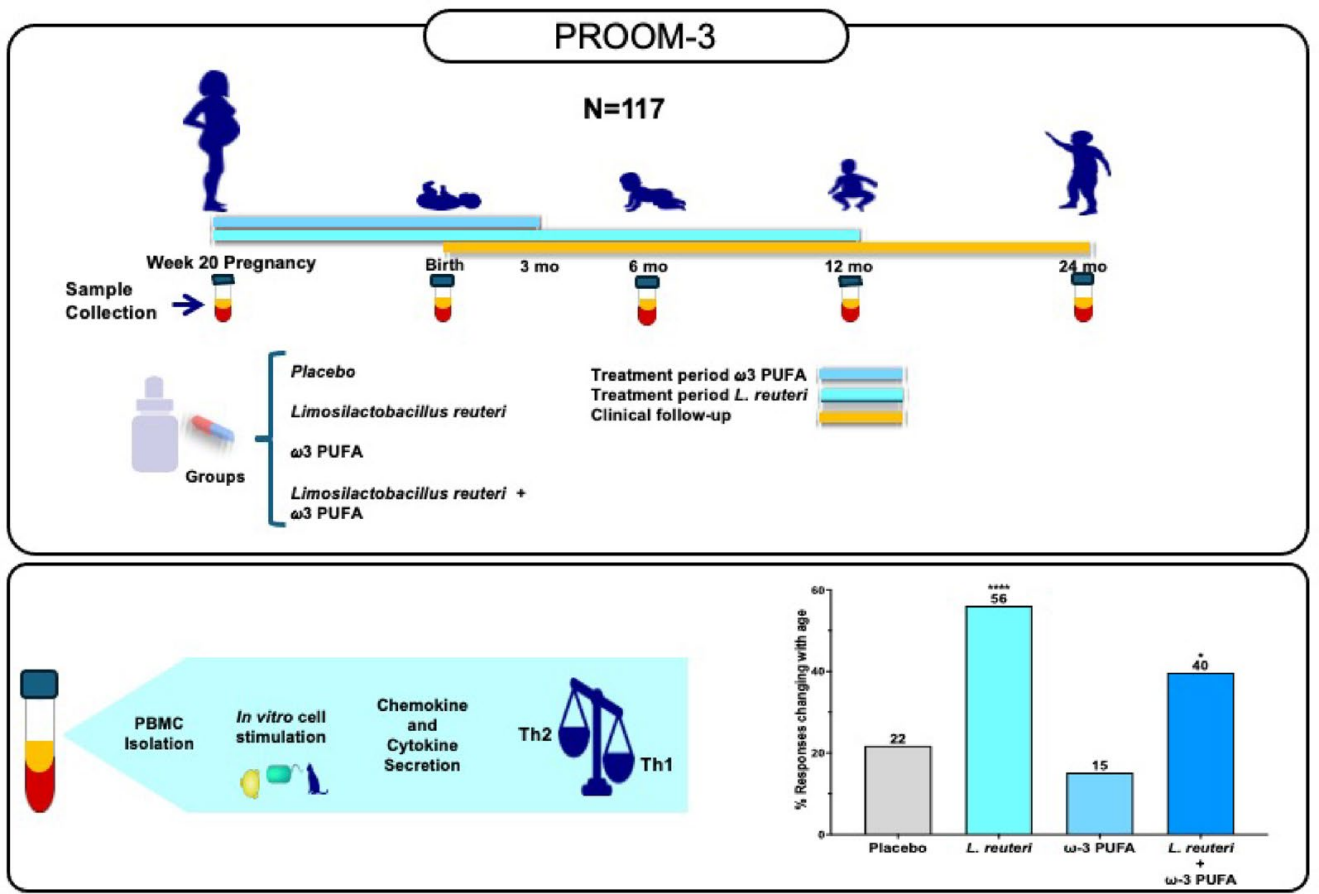
Schematic overview of the PROOM-3 study (PRObiotics and OMega-3, ClinicalTrials.gov-ID: NCT01542970). For the present study, 117 mother-baby pairs received different supplements as indicated. PBMCs from infants were analyzed *ex vivo* at several time points: 0 mo (month-old), 6 mo, 12 mo, and 24 mo. Bar graphs represent the percentage of age-dependent changes in the different supplementation groups. The results revealed that *L. reuteri* and *L. reuteri* combined with PUFA ω-3 induced more age-dependent immune changes than did the placebo, suggesting that probiotics may enhance immune maturation. Fisher’s exact test, two-sided, ****p < 0.0001; *p = 0.027

All the subjects included in this study were part of the PROOM-3 study (PRObiotics and OMega-3, ClinicalTrials.gov-ID: NCT01542970) (Fig. 1) [26, 27]. This study is a randomized, double-blind, placebo-controlled trial that is currently active and recruiting participants.

The supplementation protocol is detailed by Ahlberg et al. [28]. Briefly, the participants were women at 20 weeks of pregnancy and their subsequent offspring residing in the Östergötland region of Sweden. Pregnant women (week 20) received 10^9^ colony-forming units (CFUs) of *L. reuteri* diluted in refined coconut and peanut oils (BioGaia^®^, Stockholm, Sweden) twice a day until delivery. The infants received 10^8^ CFU (5 drops) once daily from birth until they reached one year of age with the same formulation. The placebo group for *L. reuteri* received placebo drops (refined coconut and peanut oils) during the same period. For ω-3 PUFAs supplementation, pregnant women (week 20) received 3 capsules daily of Pikasol^®^ (Orkla Health, Lund, Sweden) containing 640 mg of ω-3 PUFAs (35% EPA, 25% DHA). The placebo group received capsules containing olive oil. Infant ω-3 PUFAs supplementation was provided indirectly via breast milk until 3 mo.

For the present study, 117 infants were divided into four treatment groups: placebo, *L. reuteri*, ω-3 PUFAs and *L. reuteri* plus ω-3 PUFAs. The participants’ characteristics are shown in Table 1. Among the variables, only the presence of pets in the families of 12-month-old infants differed significantly between the placebo group and the *L. reuteri* and ω-3 PUFAs groups. The variability in the *n* (number of samples) by supplementation group and time point was due to randomization and/or sample availability.

### Peripheral blood mononuclear cell Isolation

Peripheral blood mononuclear cells (PBMCs) from infants were collected at birth (cord blood) and at 6, 12, and 24 mo and processed according to Forsberg et al. [15]. Venous and umbilical cord blood were drawn into heparin tubes and centrifuged for 10 min at 400 × g. The plasma was discarded and replaced with Roswell Park Memorial Institute (RPMI) medium (Thermo Fisher Scientific, Uppsala, Sweden), and the samples were diluted 1:2 with RPMI (1:3 for cord blood cells). PBMCs were isolated by layering the diluted blood over a Ficoll-Paque (Sigma‒Aldrich, Stockholm, Sweden) density gradient, followed by centrifugation for 30 min at 400 × g. The lymphocyte layer was collected, transferred to a fresh tube, and washed with RPMI supplemented with 2% fetal calf serum (FCS) (Gibco, Thermo Fisher Scientific, MS, USA), followed by centrifugation for 10 min at 400 × g with a low brake. This washing step was repeated three times. The cells were counted and frozen at a concentration of 5–10 × 10⁶ cells/mL in cryomedia (10% dimethyl sulfoxide [DMSO], 50% heat-inactivated FCS, and 40% RPMI) for later stimulation.

### Cell stimulations

To assess cytokine responses from both adaptive and innate immune cells, PBMCs were stimulated for 6 days with environmental allergens (birch pollen and cat), the food allergen ovalbumin and the vaccine antigen tetanus toxoid to evaluate T-cell-derived cytokine production. Phytohemagglutinin (PHA) was included as a positive control for polyclonal T-cell activation. In parallel, PBMCs were stimulated for 24 h with TLR ligands that activate innate immune cells, including lipopolysaccharide (LPS), lipoteichoic acid (LTA), and cytosine-phosphate-guanine oligodeoxynucleotides (CpGs), which specifically target TLR4, TLR2, and TLR9, respectively.

PBMCs from infants were cultured at a density of 1 × 10^6^ viable cells/mL in AIM-V medium (Thermo Fisher Scientific, Uppsala, Sweden) supplemented with 20 µM β-mercaptoethanol (β-ME, Sigma). The cells were divided into unstimulated controls (cultured in AIM-V with β-ME alone) and stimulated groups and exposed to the following antigens at final concentrations: 100 µg/mL ovalbumin (Sigma‒Aldrich, Merck, Darmstadt, Germany), 10 µg/mL birch allergen (ALK, Hørsholm, Denmark), 5 µg/mL cat allergen (ALK), 2 µg/mL PHA (Sigma‒Aldrich), 100 ng/mL tetanus toxoid (Sigma‒Aldrich), 5 ng/mL LPS (InvivoGen, Toulouse, France), 1 µg/mL LTA (InvivoGen), and 1 µg/mL CpG ODN M362 (InvivoGen).

The cells stimulated with PHA, LPS, LTA, CpG, or the unstimulated controls were incubated for 24 h at 37 °C in 5% CO_2_. For IL-4 analysis, 2 µg/mL anti-IL-4 receptor blocking antibodies (Bio-Techne, Abingdon, United Kingdom) were added to cell cultures containing ovalbumin, birch, cat, and tetanus toxoid to prevent IL-4 consumption by PBMCs. These cultures, along with their respective controls, were incubated for 6 days under the same conditions. The number of viable cells determined the number of *ex vivo* stimulations. The order of priority was as follows: ovalbumin, birch, PHA, cat, LPS, LTA, CpG and tetanus toxoid. Following incubation, all the samples were centrifuged at 400 × g for 15 min. The supernatants were collected and stored at − 70 °C for subsequent analysis.

To assess the immunomodulatory effects of probiotic supplementation on early immune maturation, we measured a panel of cytokines and chemokines representing key immune pathways (Supplemental Table 1). These included Th1-associated cytokines: Interferon gamma (IFN-γ), Interleukin (IL) -12p70, and C-X-C motif chemokine ligand 10 (CXCL10), previously known as interferon gamma induced protein 10 (IP-10) [29], Th2-associated cytokines: IL-4, IL-5, IL-13, and C-C Motif Chemokine Ligand 17 (CCL17), regulatory cytokine: IL-10, Th17-related markers: IL-17 A, IL-23, and IL-1β, and proinflammatory mediators: IL-6 and Tumor Necrosis Factor (TNF), previously called TNF-α [30]. This selection was based on their established roles in shaping immune polarization, tolerance induction, and allergic sensitization [31, 32].

The levels of cytokines and chemokines in the conditioned supernatants were determined via the Meso Scale Discovery^®^ (MSD) Multi-Spot Assay System (Rockville, MD, USA) following the manufacturer’s instructions [33]. Two customized assay panels were developed based on the U-PLEX Biomarker Group 1 Human Assays (Cat. No. K15067L-1). A 7-Plex panel was used to quantify the following analytes: IFN-γ, IL-10, IL-13, IL-17 A, IL-5, CXCL10 and CCL17. A 6-plex panel was used to quantify the levels of IL-10, IL-12p70, IL-1β, IL-23, IL-6, and TNF, specifically to evaluate the secretion of innate cytokines following stimulation with TLR ligands. Additionally, IL-4 was quantified via a V-plex Proinflammatory Panel 1 Human Kit (Cat. number K15049D). All the data were acquired on an MSD plate reader and analyzed via Discovery Workbench software (MSD, Rockville, MD, USA). Parameters for calculations, including the determination of the lower detection limit (LDL) and upper detection limit (UDL), were applied according to Numis et al. [34]. The LDL values for all analytes are provided in Supplemental Table 1. For samples with undetectable concentrations, values were imputed as half the cutoff level, as previously described [15, 34, 35]. Assay sensitivity was evaluated by calculating the frequency of values within the LDL and/or extrapolated beyond it. For all cytokine and chemokine measurements, values obtained from the stimulated conditions were subtracted from unstimulated control cultures to isolate the net response attributable to each stimulus. This approach, which was previously used [36], allows for a clearer interpretation of stimulus-specific immune responses. A total of 70 conditions were tested, and the specific cytokines and chemokines measured for each stimulation condition are detailed in Supplemental Table 1.

### Statistics

All the analyses regarding the intervention were conducted in R version 4.3.1 [37] by a blinded researcher. Continuous clinical characteristics were tested for normality via the Shapiro–Wilk test. Nonnormally distributed variables were compared across supplementation groups via the Kruskal–Wallis test, whereas discrete variables were analyzed via Fisher’s exact test and are presented as counts, with sample size indicated as *n*. For cytokine and chemokine secretion analyses, both between-group comparisons at each timepoint and longitudinal comparisons across timepoints were conducted via the Kruskal–Wallis test, followed by Dunn’s multiple comparison *post hoc* test. Longitudinal comparisons were conducted via the Kruskal‒Wallis test due to incomplete data contributions across timepoints by some participants, which precluded the use of paired measures approaches. To control for multiple comparisons, the Benjamini–Hochberg correction was applied. Statistical discrimination was set at a significance level of 0.05 (*p* < 0.05), and adjusted significance was set at q < 0.10. All data are presented as medians and interquartile ranges (IQRs) because of nonnormal distributions.

**Table 1:**
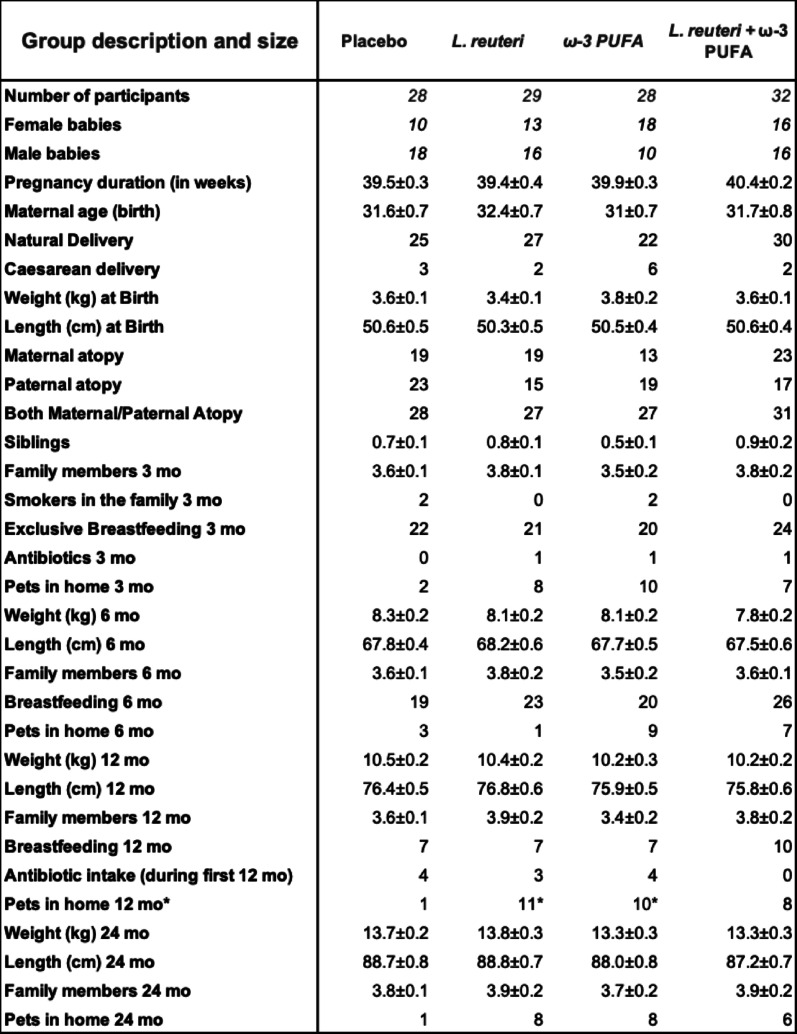
Profile of the participants. Continuous clinical characteristics are expressed as the means ± SEMs and were tested for normality via the Shapiro–Wilk test and for significance via the Kruskal–Wallis test. Discrete clinical characteristics are expressed as *n* values and were analyzed via Fisher’s exact test. * The number of families with pets when the babies were 12 mo was significantly greater in the *L. reuteri* and ω-3 groups than in the placebo group (*p* < 0,05)

## Results

Supplementation with *L. reuteri* induced significant age-dependent changes in 56% of the conditions tested (37 of 66) compared with 22% (15 of 69) in the placebo group (Fisher’s exact test, two-sided, *p* < 0.0001), indicating enhanced immune maturation after probiotic supplementation (Fig. 1, Supplemental Tables S2–S4). Furthermore, compared with placebo, double supplementation with *L. reuteri* and ω-3 PUFAs induced similar, although less pronounced, changes in 40% of the conditions (27 of 68) (Fisher’s exact test, two-sided, *p* = 0.027) (Fig. 1, Supplemental Tables S2 –S4).

**Fig. 2:**
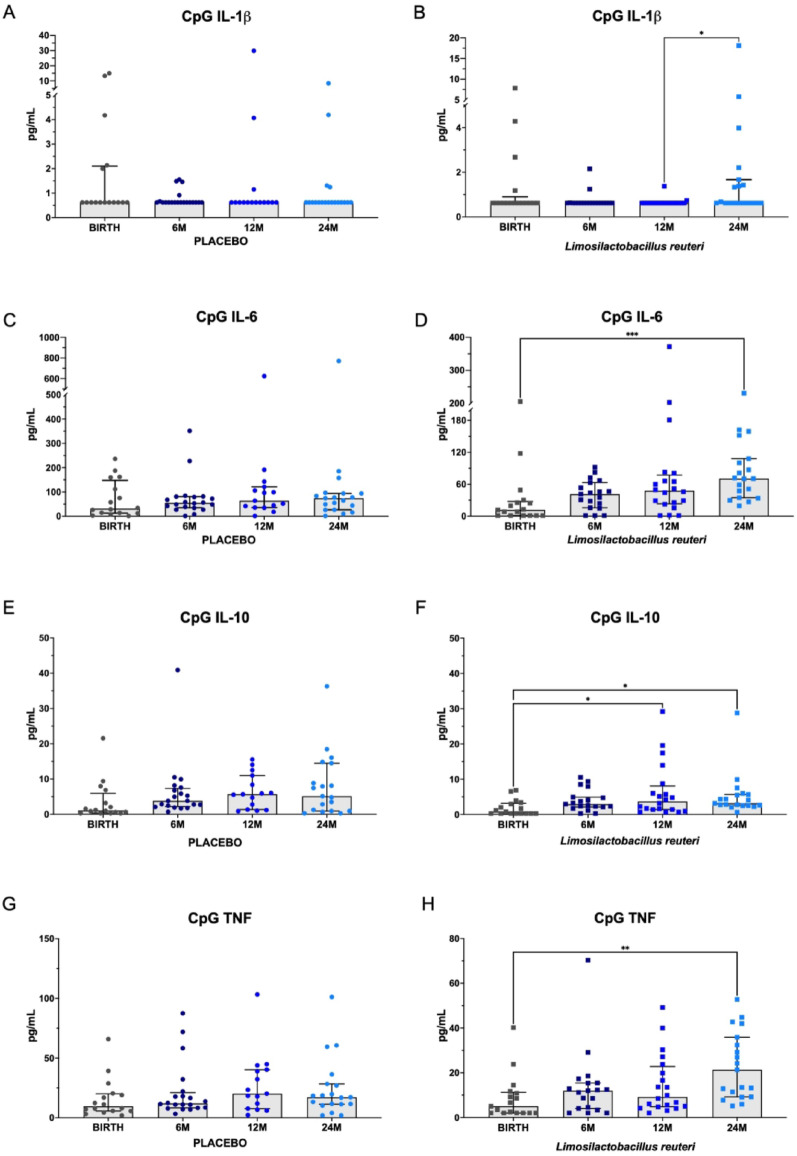
Cytokine secretion in CpG-stimulated PBMCs at birth and at 6, 12, and 24 months of age. **A** Secretion of interleukin-1β (IL-1β) in the placebo group (**A**) and the *L. reuteri* group (**B**). Secretion of interleukin-6 (IL-6) in the placebo group (**C**) and the *L. reuteri* group (**D**). Secretion of interleukin-10 (IL-10) in the placebo group (**E**) and the *L. reuteri* group (**F**). Secretion of tumor necrosis factor (TNF, previously called TNF-α) in the placebo group (**C**) and the *L. reuteri* group (**D**). The results are expressed as the median ± IQR in pg/mL. Statistical significance was evaluated via the Kruskal‒Wallis test and Dunn’s multiple comparison test for *post hoc* analysis, with *p* < 0.05 considered statistically significant. The Benjamini‒Hochberg method was applied to adjust for multiple comparisons, with statistical discrimination set at q < 0.10. The significance levels shown in the graphs are denoted as * *p* < 0.05, ** *p* < 0.01, and *** *p* < 0.001. The number of samples per group is indicated as follows: placebo: birth (*n* = 16), 6 mo (*n* = 20), 12 mo (*n* = 15), and 24 mo (*n* = 19). *L. reuteri*: Birth (*n =* 17), 6 mo (*n* = 19), 12 mo (*n* = 20), 24 mo (*n* = 19)

To illustrate immune maturation over time, responses to CpG stimulation are presented. In the placebo group, no age-dependent changes were observed in the IL-1β, IL-6, IL-10, or TNF responses (Fig. 2, Supplemental Tables S3 –S4). In contrast, the *L. reuteri-*supplemented group presented significantly greater IL-10 responses after CpG stimulation at 12 and 24 months than at birth. Additionally, IL-1β, IL-6 and TNF secretion were significantly elevated at 24 mo compared with those at birth, suggesting a long-term effect of *L. reuteri* supplementation on immune maturation. Similar effects were observed in the double‑supplementation group (*L. reuteri* and ω‑3 PUFA) after CpG stimulation, with significantly increased IL‑6 and TNF secretion at 12 and 24 months, and significantly elevated IL‑10 responses at 6, 12, and 24 months compared with birth (Supplemental Tables S3 –S4).

**Fig. 3:**
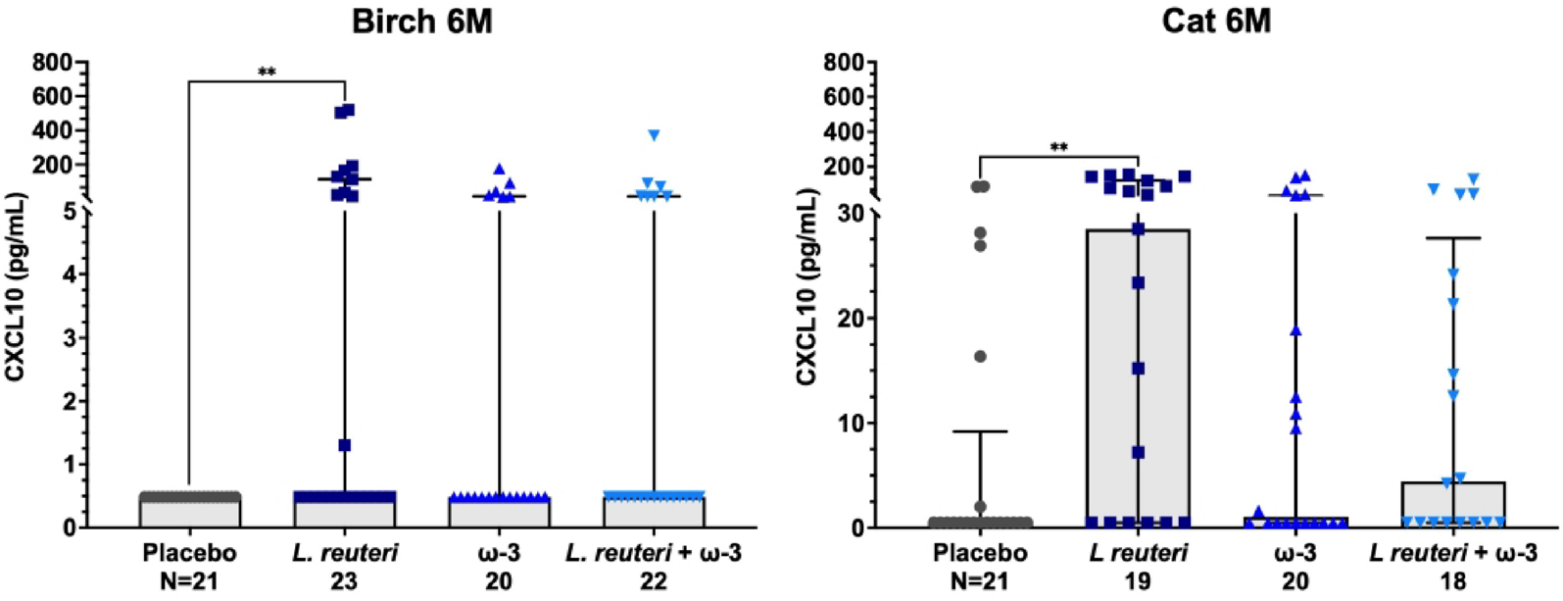
Effects of *L. reuteri*, PUFA ω-3 and combined supplementation on CXCL10 secretion in PBMCs from 6 mo infants. PBMCs were stimulated *ex vivo* with birch and cat allergens. For more details, see methods. The results are expressed as the median ± IQR in pg/mL. Statistical differences among supplementation groups were assessed via the Kruskal‒Wallis test, followed by Dunn’s multiple comparison test for *post hoc* analysis. *p* < 0.05 was considered statistically significant. The Benjamini‒Hochberg method was applied to adjust for multiple comparisons, with statistical discrimination set at q < 0.10. The significance levels shown in the scatter plots are denoted as ** *p* < 0.01. *n* as indicated below the x axes

Furthermore, analysis of supplementation effects at specific time points revealed that *L. reuteri* supplementation significantly increased Th1-associated CXCL10 levels in response to birch and cat allergens at 6 months compared with those in the placebo group (Fig. 3).

## Discussion

This study demonstrates that maternal supplementation with *L. reuteri* from gestational week 20 of pregnancy through the infant’s first year modulates allergen-induced responses during infancy. Previous clinical studies by our group and others suggest that combined pre- and postnatal supplementation with probiotics and/or ω-3 PUFAs may exert allergy-preventive effects [13, 17, 21].

Immune development and modulation during early life are critical processes through which immune cells acquire functional competence and establish tolerance to harmless antigens. These mechanisms shape an individual’s response to environmental stimuli and can lead to clinical consequences, such as the development of allergic diseases [38, 39].

The balance of innate and adaptive immune cells, including specifically Th1/Th2/Th17/Treg cell populations, determines the characteristics of the immune response and homeostasis [38, 40]. The microbiota plays a pivotal role during immune maturation in training and modulating the immune system [4, 11, 41]. Factors such as full-term birth, diet and the environment influence the development of a healthy microbiota, which modulates and determines homeostasis not only in the gut but also systemically [41].

Over the years, the benefits of probiotic supplementation for allergy prevention have been reported [18]. In previous studies from our group, supplementation with *L. reuteri* in mothers from week 36 of pregnancy and to the child during the first year of life reduced IgE-associated eczema at two years of age [17], whereas it had no effect on the plasma levels of Th1-related chemokines [42]. Furthermore, oral treatment with *L. reuteri* was able to protect mice from allergic reactions in studies using experimental models of asthma [25, 43]. Here, we observed, at 6 mo, increased CXCL10 secretion after birch and cat stimulation in the group receiving *L. reuteri* supplementation. CXCL10 is a Th1-associated chemokine that may counteract Th2-mediated allergic responses [44], and its lower plasma levels at 12 months were associated with allergen sensitization in children [42, 45]. The results presented here support our hypothesis that earlier initiation of *L. reuteri* supplementation, beginning at week 20 of pregnancy, may more effectively promote Th1 responses. Moreover, elevated CXCL10 secretion has been linked to favorable outcomes in asthmatic patients treated with omalizumab, an anti-IgE monoclonal antibody used for severe respiratory allergies and, more recently, for food allergy treatment [46]. This further underscores the potential clinical relevance of our findings.

The infant immune system undergoes rapid and dynamic changes during the first years of life, and environmental exposures in this critical window can significantly influence immune maturation and the risk of developing allergic diseases [11, 47]. Toll-like receptors (TLRs), such as TLR2, TLR4 and TLR9, play pivotal roles in this process by regulating the production of IgE, a key mediator of allergic responses. The activation of TLR9 has been shown to suppress IgE synthesis, highlighting a potential mechanism through which innate immune signaling may modulate allergic disease development [48]. Previously, a defining hallmark of nonallergic children was the initially low responsiveness of innate microbial recognition pathways—particularly TLR activity—at birth, followed by a gradual, age-related increase that parallels the maturation of the adaptive immune system, characterized by Th1 consolidation and reduced Th2 differentiation [49]. Such coordinated development suggests the importance of microbial exposure in shaping a protective immune trajectory, positioning TLRs as central regulators of both innate and adaptive immunity and key modulators in the prevention of allergic disease [49, 50].

An important observation in the present study was a temporal modulation of age-dependent changes in immune responses during infancy, under early pre- and postnatal supplementation with *L. reuteri*, compared with the placebo group: 37 vs. 15 age-dependent changes, respectively. Among those, one condition tested was stimulation with CpG, which activates TLR9 [51]. Exposure to bacterial DNA containing unmethylated CpGs may confer some protection against allergies in children living on a farm, and it has been suggested as an adjuvant in the treatment of allergic diseases [52].

In our study, compared with birth, CpG stimulation led to increased secretion of IL-1β, IL-10, IL-6, and TNF at 24 months in infants who received *L. reuteri* supplementation, indicating enhanced immune maturation. Similar effects were observed in the group receiving combined supplementation with *L. reuteri* and ω-3 PUFAs. In contrast, no significant age-dependent changes in these cytokines were observed in the placebo group. These findings support the hypothesis that *L. reuteri* supplementation during early life may positively influence immune maturation and lower the risk of allergic disease by selectively modulating innate immune pathways, including those mediated by Toll-like receptor 9 (TLR9). To reinforce the evidence for its protective role, future studies should aim to correlate these immunological profiles—particularly cytokine responses—with clinically relevant allergy outcomes.

Our results are in line with those of a murine study by Forsythe et al. [25], which observed that oral *L. reuteri* treatment attenuated asthmatic responses through TLR9 regulation, indicating that this pathway is important for the *L. reuteri* effect. Moreover, Sabatel et al. [53] reported that CpG exposure protected mice from allergic airway inflammation through an IL-10-dependent mechanism. Additionally, our results support previous studies showing that *L. reuteri* supplementation is associated not only with improved clinical outcomes but also with the modulation of IgE secretion and molecular markers, indicative of a more mature immune system [17, 27, 35, 54].

PUFAs also play a significant role in the maturation of the immune system since they are integral components of immune cell membranes and influence signaling and function [55]. A higher ω-3/ω-6 PUFAs has been associated with reduced inflammation and a lower risk of allergic reactions, particularly during early life, when the immune system is still developing [56]. A proinflammatory maternal profile during pregnancy, often linked to decreased dietary ω-3 PUFAs and increased intake of ω-6 PUFA–rich oils, may predispose offspring to various diseases [41, 57–59]. This imbalanced ratio has been shown to be related to autoimmune diseases, cardiovascular complications and allergies [12, 60]. Interventions involving ω-3 PUFA supplementation during pregnancy may modulate the gut microbiota of offspring and induce a more anti-inflammatory profile with a protective effect against pathological conditions such as allergies [20, 21, 61, 62]. In fact, supplementation during pregnancy with ω-3 PUFAs alone was associated with a decrease in IgE-associated allergic disease during infancy [21, 22].

Bearing this in mind, in the present study, we hypothesized that ω-3 PUFAs combined with *L. reuteri* would have beneficial immunomodulatory effects. However, ω-3 PUFA supplementation did not clearly affect these parameters in this study, which may reflect the distinct mechanisms through which these interventions act. The positive effect of the combined supplementation may be attributed to the immune-modulatory effects of *L. reuteri*. Our results are in accordance with a previous study from our group with PROOM-3 participants, which showed that *L. reuteri* supplementation was able to induce peripheral changes in Treg populations, whereas ω-3 PUFA supplementation had no effect [26]. While ω-3 PUFAs are well documented for their anti-inflammatory properties [56], their role in early immune development remains less clear, indicating a need for further exploration of their immunomodulatory potential.

Overall, this study provides compelling evidence that probiotic supplementation with *L. reuteri* enhances immune maturation during infancy. These findings underscore the importance of distinguishing between the immune-stimulatory properties of probiotics and the anti-inflammatory effects of ω-3 PUFAs. While probiotics may directly activate immune cells, ω-3 PUFAs likely exert their effects by modulating inflammatory pathways, potentially contributing to a more balanced immune response over time.

## Conclusions

Our study demonstrated that early pre- and postnatal intervention with *L. reuteri*, initiated at gestational week 20, supports immune maturation during infancy. Probiotic supplementation was associated with enhanced allergen-induced Th1-associated responses at 6 months of age. In contrast, supplementation with ω-3 PUFAs alone did not result in clear immunomodulatory effects. Future research will focus on linking these immunological findings to clinical allergy outcomes in infancy to further clarify the protective potential of these interventions.

## Supplementary Information

Below is the link to the electronic supplementary material.

Supplementary Material 1

## Data Availability

The data that support the findings of this study are not openly available due to reasons of sensitivity, but are available from the corresponding author upon reasonable request.

## Abbreviations

CCL17: C-C motif chemokine ligand 17
CFUs: colony-forming units
CpGs: cytosine-phosphate-guanine oligodeoxynucleotides
CXCL10: C-X-C motif chemokine ligand 10, previously called IP-10
DHA: docosahexaenoic acid
DMSO: dimethyl sulfoxide
EPA: eicosapentaenoic acid
FCS: fetal calf serum
GI: gastrointestinal
IFN-γ: Interferon gamma
IgE: Immunoglobulin E
IL: Interleukin
IQRs: interquartile ranges
*L. reuteri*: *Limosilactobacillus* *reuteri*
LDL: lower detection limit
LPS: lipopolysaccharide
LTA: lipoteichoic acid
mo: months of age
MSD: Meso Scale Discovery^®^
n: number of samples
PBMCs: Peripheral blood mononuclear cells
PHA: Phytohemagglutinin
PROOM-3: PRObiotics and OMega-3
PUFAs: polyunsaturated fatty acids
RPMI medium: Roswell Park Memorial Institute medium
Th: T helper cells
TLRs: Toll-like receptors
TNF: Tumor necrosis factor
Treg: T regulatory cells
UDL: upper detection limit
β-ME: β-mercaptoethanol
