# Supplementary material for: Effects of pre- and postnatal probiotic and ω-3 fatty acid supplementation on cytokine and chemokine responses to allergens and TLR ligands during infancy
Source: Allergy Asthma Clin Immunol. 2026 May 5;22:27. doi: 10.1186/s13223-026-01036-y (PMC13141264; doi:10.1186/s13223-026-01036-y)
Supplement: Supplementary file 1 — Supplementary Material 1 [file 13223_2026_1036_MOESM1_ESM.pdf]

Effects of pre- and postnatal probiotic and ω-3 fatty acid supplementation on cytokine and chemokine responses to allergens and TLR ligands during infancy

Cibely C Fontes-Oliveira[1], Amanda Nylén[1], Johanna Ljung[1], Astrid Welin[1], Lovisa Arvidsson[1], Magalí Martí[3], Dhanapal Govindaraj[1], Isabel García Martín[1], Camilla Janefjord[1], Lina Tingö[1], Ahmed Al-Kaabawi[1], Elisabet Severin[2,3], Karel Duchén[2,3] and Maria C Jenmalm[1,2*]

[1] Division of Inflammation and Infection, Department of Biomedical and Clinical Sciences, Linköping University, Linköping, Sweden

[2] Allergy Center, University Hospital, Linköping, Sweden

[3] Division of Children's and Women's Health, Department of Biomedical and Clinical Sciences, Linköping University, Linköping, Sweden.

***Correspondence:** Maria C Jenmalm - maria.jenmalm@liu.se

SUPPLEMENTARY INFORMATION

Tables S1-S4.

S1 - Supplemental Table 1

Overview of stimulations *in vitro* and measurements performed in this study using Mesoscale Discovery assays. The lowest detection limits of the chemokines and cytokines are indicated.

MSD	Chemo- Cytokine	Lowest detection limits (pg/mL)	Unstimulated	CpG	LPS	LTA	Birch	Cat	OVA	PHA	Tetanus
6-PLEX	IL-1β	1.24	X	X	X	X					
	IL-6	2.20	X	X	X	X					
	IL-10	0.58	X	X	X	X					
	IL-12p70	0.79	X	X	X	X					
	IL-23	9.42	X	X	X	X					
	TNF	4.02	X	X	X	X					
7-PLEX	CCL17	0.68	X				X	X	X	X	X
	CXCL10	0.97	X				X	X	X	X	X
	IFN-γ	9.94	X				X	X	X	X	X
	IL-5	1.75	X				X	X	X	X	X
	IL-10	0.20	X				X	X	X	X	X
	IL-13	4.91	X				X	X	X	X	X
	IL-17A	6.06	X				X	X	X	X	X
V-PLEX	IL-4	0.03					X	X	X		X
**Total**											**70**

S2 - Supplemental Table 2

Cytokine and chemokine secretion induced *in vitro* by birch, cat and ovalbumin allergens in PBMC from infants. Values expressed as pg/mL. Median, IQR (1st and 3rd quartile values) and N are indicated. Statistical significance was evaluated by the Kruskal Wallis (p values) test and then by the post-hoc Dunn test. Corrections for multiple comparisons were performed using the Benjamini-Hochberg method (q values). The letters a, b and c were used to express the differences among groups, and columns with the same letter are not significantly different.

			Birth				6 mo				12 mo				24 mo				Kruskal Wallis	Benjamin-Hochberg
Group	Stimuli	Chemo-Cytokine	Median	IQR 25	IQR 75	N	Median	IQR 25	IQR 75	N	Median	IQR 25	IQR 75	N	Median	IQR 25	IQR 75	N	p values	q values
Placebo	Birch	CCL17	0.34	0.34	1.43	16	0.34	0.34	1.19	21	7.72	0.34	62.86	17	11.30	0.34	102.24	22	0.023	0.138
		CXCL10	0.49	0.49	0.49	16	0.49	0.49	0.49	21	0.49	0.49	16.01	17	0.49	0.49	116.11	22	0.090	0.184
		IFN-γ	4.97[a]	4.97	42.28	16	59.57[ab]	4.97	137.07	21	96.46[b]	9.88	286.87	17	248.02[b]	21.75	1148.06	22	0.001	0.006
		IL-4	0.01	0.01	0.06	15	0.04	0.01	0.05	14	0.03	0.01	0.12	9	0.06	0.01	0.24	10	0.324	0.326
		IL-5	0.87[a]	0.87	0.87	16	0.87[a]	0.87	0.87	21	0.87[ab]	0.87	5.34	17	2.77[b]	0.87	10.33	22	0.002	0.006
		IL-10	0.10[a]	0.10	0.10	16	0.10[a]	0.10	0.10	21	0.10[ab]	0.10	0.19	17	0.25[b]	0.10	0.78	22	0.010	0.020
		IL-13	2.45	2.45	2.45	16	2.45	2.45	4.28	21	2.45	2.45	18.54	17	4.41	2.45	19.37	22	0.049	0.122
		IL-17A	3.03	3.03	3.03	16	3.03	3.03	3.03	21	3.03	3.03	3.03	17	3.03	3.03	13.11	22	0.074	0.222
	Cat	CCL17	5.68	0.34	44.47	16	4.21	0.34	47.69	21	10.71	2.41	26.63	16	5.20	0.34	48.00	22	0.821	0.898
		CXCL10	6.98	0.49	657.09	16	0.49	0.49	9.18	21	24.56	1.35	62.43	16	0.49	0.49	2.74	22	0.019	0.114
		IFN-γ	8.59	4.97	45.62	16	10.96	4.97	149.04	21	68.60	4.97	142.15	16	20.05	4.97	169.47	22	0.274	0.329
		IL-4	0.01	0.01	0.08	15	0.03	0.01	0.07	12	0.04	0.03	0.08	8	0.05	0.02	0.42	4	0.326	0.326
		IL-5	0.87	0.87	0.87	16	0.87	0.87	0.87	21	0.87	0.87	2.83	16	0.87	0.87	1.17	22	0.281	0.281
		IL-10	0.10[a]	0.10	0.10	16	0.10[ab]	0.10	0.14	21	0.17[bc]	0.10	0.22	16	0.24[c]	0.10	0.65	22	0.000	0.000
		IL-13	2.45	2.45	4.92	16	2.45	2.45	6.09	21	2.45	2.45	6.59	16	2.45	2.45	3.77	22	0.985	0.985
		IL-17A	3.03	3.03	3.03	16	3.03	3.03	3.03	21	3.03	3.03	3.03	16	3.03	3.03	3.03	22	0.467	0.477
	OVA	CCL17	12.09	2.53	35.69	16	3.29	0.34	86.73	21	37.47	2.86	164.64	17	40.77	0.57	498.68	22	0.329	0.494
		CXCL10	22.66	0.49	731.18	16	56.71	9.67	266.07	21	58.69	9.25	143.55	17	3.06	0.49	127.19	22	0.381	0.381
		IFN-γ	350.47[a]	162.11	1343.68	16	3016.41[b]	898.67	11303.93	21	2701.69[b]	1457.55	8777.95	17	6107.36[b]	1 164.05	8953.81	22	0.003	0.009
		IL-4	0.03	0.01	0.08	16	0.06	0.03	0.19	15	0.08	0.06	0.13	9	0.08	0.03	0.26	12	0.198	0.326
		IL-5	7.69	2.01	15.07	16	5.25	0.87	51.04	21	17.46	3.96	53.08	17	15.60	0.87	208.81	22	0.226	0.271
		IL-10	1.26	0.11	3.48	16	0.64	0.14	1.02	21	0.83	0.41	1.94	17	0.95	0.37	3.50	22	0.235	0.294
		IL-13	5.38	2.45	16.34	16	7.74	3.71	22.01	21	10.01	5.00	31.92	17	12.49	2.45	86.02	22	0.294	0.353
		IL-17A	4.60	3.03	17.64	16	10.28	3.03	20.52	21	13.78	3.03	40.17	17	11.85	3.03	69.97	22	0.352	0.477
L. reuteri	Birch	CCL17	0.34[a]	0.34	0.44	17	0.34[ab]	0.34	4.23	23	1.71[ab]	0.34	25.62	22	5.64[b]	0.34	61.61	22	0.015	0.042
		CXCL10	0.49	0.49	44.79	17	0.49	0.49	114.54	23	0.49	0.49	2.70	22	0.49	0.49	111.98	22	0.436	0.557
		IFN-γ	4.97[a]	4.97	25.80	17	25.48[ab]	4.97	349.74	23	31.34[ab]	4.97	214.33	22	111.92[b]	6.20	470.73	22	0.022	0.033
		IL-4	0.04	0.01	0.06	17	0.03	0.01	0.06	14	0.04	0.01	0.09	15	0.09	0.01	0.18	13	0.334	0.334
		IL-5	0.87	0.87	0.87	17	0.87	0.87	0.87	23	0.87	0.87	2.92	22	0.87	0.87	8.95	22	0.052	0.078
		IL-10	0.10[a]	0.10	0.10	17	0.10[ab]	0.10	0.10	23	0.10[ab]	0.10	0.18	22	0.10[b]	0.10	0.28	22	0.040	0.067
		IL-13	2.45[a]	2.45	2.45	17	2.45[ab]	2.45	5.43	23	2.45[ab]	2.45	6.13	22	5.46[b]	2.45	10.02	22	0.005	0.015
		IL-17A	3.03[a]	3.03	3.03	17	3.03[a]	3.03	3.03	23	3.03[ab]	3.03	3.03	22	3.03[b]	3.03	15.90	22	0.001	0.003
	Cat	CCL17	0.43[a]	0.34	1.82	17	15.22[b]	1.32	65.18	21	6.95[ab]	0.34	20.35	19	16.28[b]	1.21	61.30	20	0.007	0.042
		CXCL10	7.29	0.49	58.09	17	23.33	0.49	104.64	21	0.49	0.49	77.43	19	20.47	0.49	129.32	20	0.464	0.557
		IFN-γ	4.97	4.97	22.20	17	21.95	4.97	322.65	21	157.10	4.97	597.87	20	61.88	4.97	281.33	19	0.044	0.053
		IL-4	0.01[a]	0.01	0.02	17	0.04[ab]	0.01	0.07	13	0.06[ab]	0.01	0.11	14	0.08[b]	0.01	0.17	11	0.013	0.026
		IL-5	0.87	0.87	0.87	17	0.87	0.87	1.14	21	0.87	0.87	0.87	19	0.87	0.87	1.64	20	0.752	0.752
		IL-10	0.10[a]	0.10	0.10	17	0.10[ab]	0.10	0.30	21	0.19[b]	0.10	0.40	19	0.21[b]	0.10	0.45	20	0.000	0.000
		IL-13	2.45	2.45	2.45	17	2.45	2.45	5.59	21	2.45	2.45	3.54	19	2.45	2.45	4.14	20	0.368	0.442
		IL-17A	3.03	3.03	3.03	17	3.03	3.03	3.03	21	3.03	3.03	3.03	19	3.03	3.03	3.03	21	0.044	0.048
	OVA	CCL17	12.25	3.42	39.68	17	32.66	11.08	85.22	23	46.65	2.20	185.40	22	50.54	0.34	239.13	22	0.370	0.370
		CXCL10	11.97	0.49	190.27	17	19.02	0.49	110.57	23	25.98	0.49	151.89	22	15.41	0.49	142.48	22	0.923	0.923
		IFN-γ	147.63[a]	47.51	1198.74	17	1532.98[ab]	379.04	3123.74	23	3827.16[b]	1288.36	7090.14	22	3116.38[b]	581.01	6476.76	22	0.004	0.008
		IL-4	0.01	0.01	0.05	17	0.06	0.01	0.14	15	0.04	0.01	0.15	17	0.09	0.05	0.12	15	0.071	0.095
		IL-5	1.81	0.87	14.85	17	9.14	0.87	40.07	23	19.15	1.34	55.40	22	11.40	1.86	36.81	22	0.137	0.164
		IL-10	0.79	0.15	1.09	17	1.05	0.27	1.52	23	0.91	0.20	1.98	22	0.94	0.17	2.10	22	0.760	0.760
		IL-13	2.45	2.45	13.84	17	13.51	2.45	23.62	23	15.26	2.45	31.05	22	12.04	2.45	26.09	22	0.052	0.078
		IL-17A	3.03[a]	3.03	13.06	17	17.31[b]	6.23	45.41	23	22.65[b]	3.03	56.37	22	23.99[b]	5.49	38.76	22	0.019	0.028

			Birth				6 mo				12 mo				24 mo				Kruskal Wallis	Benjamin-Hochberg
Group	Stimuli	Chemo-Cytokine	Median	IQR 25	IQR 75	N	Median	IQR 25	IQR 75	N	Median	IQR 25	IQR 75	N	Median	IQR 25	IQR 75	N	p values	q values
ω-3	Birch	CCL17	0.34	0.34	169.05	19	1.87	0.34	35.56	20	0.66	0.34	44.66	21	1.58	0.34	57.67	21	0.988	0.988
		CXCL10	0.49	0.49	320.68	19	0.49	0.49	14.12	20	0.49	0.49	15.72	21	0.49	0.49	0.49	21	0.196	0.442
		IFN-γ	8.53	4.97	459.30	19	53.81	4.97	293.52	19	86.62	4.97	667.20	21	30.73	4.97	873.79	21	0.630	0.630
		IL-4	0.01	0.01	0.07	16	0.05	0.01	0.13	10	0.06	0.03	0.13	13	0.02	0.01	0.11	10	0.408	0.408
		IL-5	0.87	0.87	1.04	19	0.87	0.87	1.91	20	0.87	0.87	2.69	21	2.31	0.87	7.32	21	0.207	0.310
		IL-10	0.10	0.10	0.16	19	0.10	0.10	0.10	20	0.10	0.10	0.20	21	0.11	0.10	0.20	21	0.158	0.370
		IL-13	2.45	2.45	13.10	19	2.45	2.45	6.69	20	2.45	2.45	8.37	21	2.45	2.45	14.05	21	0.784	0.941
		IL-17A	3.03	3.03	3.03	19	3.03	3.03	3.03	21	3.03	3.03	3.03	21	3.03	3.03	3.03	20	0.962	0.962
	Cat	CCL17	4.06	0.34	25.33	19	7.75	0.62	122.54	20	55.09	2.72	270.77	20	26.12	0.34	123.11	21	0.394	0.788
		CXCL10	0.49	0.49	584.80	19	1.05	0.49	31.81	20	15.57	0.49	65.30	20	0.92	0.49	42.18	21	0.906	0.906
		IFN-γ	4.97	4.97	94.75	19	35.04	4.97	234.46	19	159.25	25.05	487.00	20	23.12	4.97	603.08	21	0.089	0.178
		IL-4	0.03	0.01	0.06	15	0.05	0.01	0.06	10	0.09	0.04	0.22	10	0.03	0.01	0.16	9	0.170	0.227
		IL-5	0.87	0.87	0.87	19	0.87	0.87	0.87	20	0.87	0.87	4.39	20	0.87	0.87	1.56	21	0.359	0.359
		IL-10	0.10[a]	0.10	0.14	19	0.10[a]	0.10	0.25	20	0.25[b]	0.14	0.49	20	0.19[ab]	0.10	0.47	21	0.004	0.040
		IL-13	2.45	2.45	5.29	19	2.45	2.45	6.93	20	6.53	2.45	13.36	20	2.45	2.45	6.81	21	0.189	0.284
		IL-17A	3.03	3.03	3.03	19	3.03	3.03	3.03	21	3.03	3.03	3.03	20	3.03	3.03	3.03	20	0.954	0.962
	OVA	CCL17	7.30[a]	0.34	24.20	19	43.15[ab]	11.86	106.03	20	98.18[b]	13.04	278.47	21	21.91[ab]	2.73	116.92	21	0.012	0.069
		CXCL10	4.07	0.49	263.87	19	65.74	1.82	125.07	20	37.21	6.19	196.91	21	1.10	0.49	89.44	21	0.295	0.442
		IFN-γ	831.13	151.81	3896.61	19	3164.50	988.55	9619.88	19	5372.12	1018.14	10625.68	21	3871.29	473.50	7598.59	21	0.048	0.144
		IL-4	0.05	0.02	0.09	16	0.10	0.04	0.24	12	0.16	0.06	0.27	13	0.04	0.01	0.14	11	0.052	0.104
		IL-5	9.72	0.87	18.43	19	14.66	3.52	41.19	20	18.21	9.77	63.63	21	10.53	0.87	51.00	21	0.188	0.310
		IL-10	0.95	0.38	3.56	19	0.37	0.16	1.53	20	0.86	0.15	2.89	21	1.12	0.31	2.58	21	0.486	0.694
		IL-13	7.57	2.45	19.71	19	11.23	2.45	15.85	20	29.10	7.88	42.51	21	12.13	2.45	25.68	21	0.104	0.208
		IL-17A	9.60	3.03	26.69	19	9.32	3.03	37.53	21	11.58	3.03	96.03	21	7.91	3.03	29.12	20	0.640	0.962
L. reuteri + ω-3	Birch	CCL17	0.34	0.34	35.42	19	1.65	0.34	21.67	22	2.58	0.34	40.38	21	19.40	2.80	55.49	28	0.085	0.162
		CXCL10	1.52	0.49	30.60	19	0.49	0.49	13.06	22	0.49	0.49	5.08	21	17.47	0.49	83.63	28	0.064	0.152
		IFN-γ	11.94[a]	4.97	193.86	19	48.61[a]	4.97	562.09	22	98.30[ab]	16.92	248.40	21	440.52[b]	128.86	1445.72	28	0.001	0.002
		IL-4	0.01	0.01	0.13	18	0.01	0.01	0.05	11	0.06	0.04	0.09	13	0.06	0.03	0.09	11	0.087	0.095
		IL-5	0.87	0.87	1.30	19	0.87	0.87	2.59	22	0.87	0.87	1.73	21	2.09	0.87	12.09	28	0.036	0.054
		IL-10	0.10[a]	0.10	0.10	19	0.10[ab]	0.10	0.21	22	0.10[a]	0.10	0.10	21	0.18[b]	0.10	0.33	28	0.005	0.010
		IL-13	2.45	2.45	3.41	19	2.45	2.45	5.64	22	2.45	2.45	7.99	21	4.91	2.45	11.95	28	0.071	0.132
		IL-17A	3.03	3.03	3.03	19	3.03	3.03	3.94	22	3.03	3.03	5.24	21	3.03	3.03	3.03	28	0.115	0.138
	Cat	CCL17	1.49	0.34	71.20	20	11.28	2.74	141.26	18	13.37	0.88	62.50	20	29.65	6.06	115.93	28	0.284	0.341
		CXCL10	2.65	0.49	69.84	20	4.44	0.49	27.60	18	18.33	0.49	72.93	20	26.10	0.49	75.92	28	0.517	0.517
		IFN-γ	5.51	4.97	73.78	20	39.47	4.97	240.68	18	48.96	4.97	307.70	20	94.98	4.97	596.68	28	0.164	0.164
		IL-4	0.01	0.01	0.06	18	0.04	0.01	0.05	8	0.05	0.04	0.07	11	0.05	0.03	0.11	9	0.095	0.095
		IL-5	0.87[ab]	0.87	0.87	20	0.87[a]	0.87	0.87	18	0.87[ab]	0.87	1.52	20	2.37[b]	0.87	3.77	28	0.003	0.006
		IL-10	0.10[a]	0.10	0.11	20	0.10[ab]	0.10	0.40	18	0.11[ab]	0.10	0.46	20	0.27[b]	0.16	0.69	28	0.002	0.007
		IL-13	2.45	2.45	6.26	20	3.38	2.45	5.92	18	2.45	2.45	6.54	20	2.48	2.45	7.28	28	0.670	0.804
		IL-17A	3.03	3.03	3.03	20	3.03	3.03	3.03	18	3.03	3.03	3.03	20	3.03	3.03	3.03	28	0.524	0.524
	OVA	CCL17	10.30	0.34	93.01	19	35.97	14.92	403.43	22	33.15	9.15	189.94	23	44.71	4.62	429.12	28	0.062	0.162
		CXCL10	5.74	0.49	171.96	19	89.17	0.49	151.98	22	13.64	0.49	194.31	23	141.74	4.75	291.32	28	0.160	0.240
		IFN-γ	551.96[a]	27.09	1996.33	19	4053.19[b]	1320.92	13018.64	22	2768.30[b]	1195.76	14307.45	23	5319.56[b]	1320.80	16116.58	28	0.001	0.002
		IL-4	0.02[a]	0.01	0.09	18	0.04[ab]	0.01	0.21	10	0.10[b]	0.07	0.18	15	0.10[ab]	0.06	0.17	11	0.023	0.046
		IL-5	3.12	0.87	30.27	19	23.42	3.51	76.36	22	22.49	6.44	46.92	23	15.94	6.90	83.96	28	0.175	0.210
		IL-10	0.39	0.10	0.91	19	0.75	0.25	2.15	22	0.58	0.26	2.04	23	1.07	0.29	1.48	28	0.211	0.234
		IL-13	5.39	2.45	20.82	19	20.01	6.38	45.48	22	13.40	3.08	43.07	23	13.67	3.85	43.45	28	0.088	0.132
		IL-17A	3.03	3.03	21.90	19	17.19	8.20	73.39	22	19.24	11.94	38.87	23	11.16	3.03	37.50	28	0.054	0.108

S3 - Supplemental Table 3

Cytokine secretion induced *in vitro* by CpG, LPS and LTA in PBMC from infants. Values expressed as pg/mL. Median, IQR (1st and 3rd quartile values) and N are indicated. Statistical significance was evaluated by the Kruskal Wallis (p values) test and then by the post-hoc Dunn test. Corrections for multiple comparisons were performed using the Benjamini-Hochberg method (q values). The letters a, b and c were used to express the differences among groups, and columns with the same letter are not significantly different. CpG and LTA induced IL-23 responses and IL-12p70 secretion after all stimulations are not shown, as those levels were mostly undetectable.

			Birth				6 mo				12 mo				24 mo				Kruskal Wallis	Benjamin-Hochberg
Group	Stimuli	Cytokine	Median	IQR 25	IQR 75	N	Median	IQR 25	IQR 75	N	Median	IQR 25	IQR 75	N	Median	IQR 25	IQR 75	N	p values	q values
Placebo	CpG	IL-1β	0.62	0.62	2.10	16	0.62	0.62	0.65	20	0.62	0.62	0.62	15	0.62	0.62	0.62	19	0.779	0.878
		IL-6	31.44	12.69	147.81	16	55.09	55.09	80.65	20	63.94	35.62	121.09	15	74.13	26.74	93.96	19	0.585	0.655
		IL-10	1.11	0.37	5.96	16	3.82	2.20	7.37	20	5.69	1.36	11.00	15	5.14	0.93	14.48	19	0.062	0.103
		TNF	9.59	5.66	20.06	16	11.97	11.97	21.03	20	20.19	7.68	40.29	15	17.17	11.34	28.35	19	0.292	0.389
	LPS	IL-1β	7.59	2.42	26.33	16	12.83	12.83	29.56	19	14.22	6.24	49.91	18	22.53	8.60	73.23	20	0.153	0.612
		IL-6	385.11	108.36	876.10	16	386.69	386.69	901.61	19	502.37	182.47	1 479.14	18	859.44	226.61	1731.11	20	0.368	0.655
		IL-10	0.45[a]	0.29	0.99	16	1.60[ab]	1.60	3.10	19	2.60[ab]	0.29	4.87	18	5.05[b]	0.59	11.68	20	0.002	0.005
		IL-23	4.71[a]	4.71	4.71	16	11.84[b]	11.84	18.05	19	9.54[b]	4.71	24.66	18	15.81[b]	4.71	62.77	20	0.002	0.008
		TNF	26.66[a]	13.10	68.03	16	68.93[ab]	68.93	223.62	19	87.97[ab]	47.05	248.04	18	147.92[b]	46.62	337.44	20	0.017	0.068
	LTA	IL-1β	5.14	1.34	13.58	16	4.19	4.19	12.25	19	6.19	2.75	19.51	17	3.26	1.80	10.70	20	0.674	0.878
		IL-6	331.76	64.18	909.81	16	169.62	169.62	261.60	19	135.39	58.50	758.62	17	137.64	86.71	517.45	20	0.655	0.655
		IL-10	0.29	0.29	0.45	16	0.29	0.29	1.41	19	0.65	0.29	2.61	17	0.29	0.29	1.13	20	0.378	0.420
		TNF	19.98	4.17	41.89	16	17.37	17.37	63.37	19	23.39	8.69	88.32	17	22.15	10.22	40.98	20	0.869	0.869
L. reuteri	CpG	IL-1β	0.62[ab]	0.62	0.90	17	0.62[ab]	0.62	0.62	19	0.62[a]	0.62	0.62	20	0.62[b]	0.62	1.67	19	0.023	0.092
		IL-6	11.68[a]	1.10	27.71	17	41.33[ab]	41.33	63.04	19	47.69[ab]	23.22	77.00	20	70.51[b]	34.60	107.98	19	0.001	0.004
		IL-10	0.76[a]	0.29	3.18	17	2.95[ab]	2.95	4.93	19	3.69[b]	1.39	8.07	20	3.18[b]	2.53	5.68	19	0.009	0.022
		TNF	5.02[a]	2.01	11.27	17	11.99[ab]	11.99	15.43	19	9.20[ab]	4.81	22.80	20	21.36[b]	9.23	35.87	19	0.003	0.012
	LPS	IL-1β	6.94	3.89	12.94	17	20.70	20.70	32.61	19	15.49	4.11	46.76	20	15.45	6.09	43.07	20	0.119	0.238
		IL-6	363.42	194.75	854.94	17	627.38	627.38	1437.29	19	449.26	116.30	1 210.83	20	685.14	158.26	1469.68	20	0.674	0.674
		IL-10	0.29[a]	0.29	0.75	17	1.38[ab]	1.38	3.24	19	1.39[ab]	0.29	6.94	20	2.17[b]	0.56	7.03	20	0.012	0.024
		IL-23	4.71[a]	4.71	4.71	17	4.71[ab]	4.71	31.30	19	8.98[b]	4.71	58.09	20	4.71[ab]	4.71	35.87	20	0.025	0.050
		TNF	31.02[a]	10.71	59.95	17	96.15[b]	96.15	411.42	19	67.95[ab]	17.58	211.09	20	95.26[ab]	23.99	204.82	20	0.039	0.078
	LTA	IL-1β	4.09	1.69	6.74	17	5.44	5.44	18.97	18	5.60	1.95	16.71	20	6.83	5.50	9.86	19	0.442	0.589
		IL-6	380.88	149.97	867.18	17	192.54	192.54	730.39	18	202.53	87.18	701.82	20	382.51	199.07	604.43	19	0.563	0.674
		IL-10	0.29	0.29	1.26	17	0.29	0.29	1.04	18	0.56	0.29	1.34	20	1.17	0.29	1.45	19	0.203	0.254
		TNF	22.87	17.74	40.24	17	29.42	29.42	90.02	18	22.95	8.51	65.90	20	27.59	20.57	50.99	19	0.829	0.829
ω-3	CpG	IL-1β	0.62	0.62	3.82	19	0.62	0.62	0.91	16	0.62	0.62	0.71	17	0.62	0.62	1.43	19	0.699	0.963
		IL-6	26.45	4.45	98.52	19	76.22	76.22	141.01	16	98.89	20.46	129.53	17	43.14	17.66	78.97	19	0.241	0.482
		IL-10	2.41	0.29	4.80	19	3.88	3.88	9.17	16	5.22	1.82	13.05	17	3.22	0.80	8.33	19	0.102	0.340
		TNF	11.34	6.48	26.99	19	16.28	16.28	23.11	16	15.62	10.07	31.39	17	13.38	7.55	27.13	19	0.733	0.733
	LPS	IL-1β	9.84	5.33	39.23	19	13.46	13.46	35.95	16	21.27	4.25	61.64	17	22.77	4.02	42.49	20	0.963	0.963
		IL-6	437.17	260.85	1799.63	19	329.26	329.26	1111.35	16	713.34	166.48	2721.20	17	668.43	92.10	1546.82	20	0.857	0.956
		IL-10	0.97	0.29	3.88	19	0.76	0.76	5.67	16	3.81	0.29	8.32	17	2.50	0.29	7.11	20	0.317	0.528
		IL-23	4.71	4.71	11.21	19	4.71	4.71	55.30	16	22.03	4.71	70.72	17	14.08	4.71	34.09	20	0.039	0.117
		TNF	45.65	16.61	112.51	19	69.35	69.35	223.85	16	92.13	16.56	373.49	17	89.94	19.14	214.73	20	0.498	0.733
	LTA	IL-1β	8.04	3.95	18.76	19	5.24	5.24	9.95	16	5.75	2.34	11.53	17	5.97	2.11	10.25	20	0.593	0.963
		IL-6	378.89	154.39	1 207.21	19	245.21	245.21	446.90	16	122.42	25.87	324.48	17	269.21	85.41	495.56	20	0.150	0.482
		IL-10	0.29	0.29	1.06	19	0.44	0.44	0.90	16	0.29	0.29	0.97	17	0.76	0.29	1.31	20	0.754	0.898
		TNF	17.48	8.05	40.36	19	16.84	16.84	39.95	16	23.72	7.62	26.10	17	30.00	14.61	59.82	20	0.668	0.733
L. reuteri + ω-3	CpG	IL-1β	0.62	0.62	1.71	19	0.62	0.62	0.62	17	0.62	0.62	0.62	18	0.62	0.62	0.92	23	0.101	0.202
		IL-6	11.71[a]	1.10	28.36	19	34.94[ab]	34.94	72.22	17	80.01[b]	38.78	96.24	18	57.76[b]	23.65	118.19	23	0.001	0.004
		IL-10	0.65[a]	0.29	2.12	19	3.64[b]	3.64	7.47	17	6.22[b]	2.27	9.57	18	4.51[b]	2.12	9.14	23	0.000	0.000
		TNF	7.47[a]	5.38	11.46	19	12.94[ab]	12.94	27.46	17	19.70[b]	13.78	25.05	18	22.45[b]	12.00	30.19	23	0.000	0.000
	LPS	IL-1β	5.73	1.53	13.32	20	24.65	24.65	40.44	19	36.41	5.03	94.29	18	15.38	2.74	81.05	28	0.067	0.202
		IL-6	302.99	54.00	543.95	20	460.36	460.36	1166.47	19	867.49	168.65	3 337.40	18	463.73	89.78	1346.01	28	0.182	0.182
		IL-10	0.29[a]	0.29	0.29	20	0.79[ab]	0.79	6.17	19	4.04[b]	0.59	12.02	18	1.83[b]	0.29	8.98	28	0.001	0.005
		IL-23	4.71[a]	4.71	4.71	20	4.71[ab]	4.71	116.89	19	15.35[b]	4.71	137.00	18	13.09[b]	4.71	117.40	28	0.003	0.009
		TNF	21.21[a]	7.86	36.71	20	99.13[ab]	99.13	259.35	19	164.73[b]	31.22	614.54	18	99.82[ab]	18.76	382.23	28	0.008	0.016
	LTA	IL-1β	2.98	1.40	6.02	20	3.56	3.56	5.97	18	4.51	2.48	17.63	18	4.40	0.62	12.21	27	0.428	0.428
		IL-6	215.53	54.89	444.83	20	70.62	70.62	150.13	18	250.76	53.43	668.11	18	114.76	43.71	452.66	27	0.142	0.182
		IL-10	0.29[a]	0.29	0.29	20	0.29[a]	0.29	0.29	18	0.44[ab]	0.29	3.85	18	0.64[b]	0.29	1.61	27	0.004	0.010
		TNF	9.13[a]	6.27	19.09	20	13.46[ab]	13.46	22.89	18	30.07[b]	15.69	107.99	18	15.71[ab]	11.14	30.41	27	0.015	0.020

S4 - Supplemental Table 4

Results represent age dependent differences in cytokine and chemokine secretion in PBMC from infants in the four supplementation groups and stimulated *in vitro* as indicated. p values were obtained from the Kruskal-Wallis non-parametric test. q values were obtained from the Benjamini-Hochberg method, to correct for multiple comparisons.

Stimulus	Cytokine / Chemokine	Kruskal Wallis	Benjamin-Hochberg	Kruskal Wallis	Benjamin-Hochberg	Kruskal Wallis	Benjamin-Hochberg	Kruskal Wallis	Benjamin-Hochberg
		p values Placebo	q values Placebo	p values *L reuteri*	q values *L reuteri*	p values ω-3 PUFA	q values ω-3 PUFA	p values *L reuteri* + ω-3 PUFA	q values *L reuteri* + ω-3 PUFA
No stimulation	CCL17	0.295	0.494	0.043	0.064	0.891	0.988	0.108	0.162
	CXCL10	0.248	0.298	0.285	0.557	0.518	0.622	0.076	0.152
	IFN-γ	0.014	0.028	0.001	0.003	0.342	0.451	0.004	0.006
	IL-1β	0.878	0.878	0.589	0.589	0.787	0.963	0.222	0.296
	IL-5	0.151	0.226	0.000	0.000	0.351	0.359	0.451	0.451
	IL-6	0.650	0.655	0.624	0.674	0.956	0.956	0.110	0.182
	IL-10	0.002	0.005	0.000	0.000	0.044	0.220	0.007	0.012
	IL-12p70	0.446	0.595	0.415	0.622	NA	NA	0.245	0.490
	IL-13	0.047	0.122	0.027	0.054	0.969	0.969	0.829	0.829
	IL-17A	0.477	0.477	0.048	0.048	0.954	0.962	0.044	0.108
	IL-23	0.145	0.290	NA	NA	0.471	0.634	0.305	0.305
	TNF	0.216	0.389	0.187	0.249	0.463	0.733	0.132	0.132
Birch	CCL17	0.023	0.138	0.015	0.042	0.988	0.988	0.085	0.162
	CXCL10	0.090	0.184	0.436	0.557	0.196	0.442	0.064	0.152
	IFN-γ	0.001	0.006	0.022	0.033	0.630	0.630	0.001	0.002
	IL4	0.324	0.326	0.334	0.334	0.408	0.408	0.087	0.095
	IL-5	0.002	0.006	0.052	0.078	0.207	0.310	0.036	0.054
	IL-10	0.010	0.020	0.040	0.067	0.158	0.370	0.005	0.010
	IL-13	0.049	0.122	0.005	0.015	0.784	0.941	0.071	0.132
	IL-17A	0.074	0.222	0.001	0.003	0.962	0.962	0.115	0.138
Cat	CCL17	0.821	0.898	0.007	0.042	0.394	0.788	0.284	0.341
	CXCL10	0.019	0.114	0.464	0.557	0.906	0.906	0.517	0.517
	IFN-γ	0.274	0.329	0.044	0.053	0.089	0.178	0.164	0.164
	IL-4	0.326	0.326	0.013	0.026	0.170	0.227	0.095	0.095
	IL-5	0.281	0.281	0.752	0.752	0.359	0.359	0.003	0.006
	IL-10	0.000	0.000	0.000	0.000	0.004	0.040	0.002	0.007
	IL-13	0.985	0.985	0.368	0.442	0.189	0.284	0.670	0.804
	IL-17A	0.467	0.477	0.044	0.048	0.954	0.962	0.524	0.524
CpG	IL-1β	0.779	0.878	0.023	0.092	0.699	0.963	0.101	0.202
	IL-6	0.585	0.655	0.001	0.004	0.241	0.482	0.001	0.004
	IL-10	0.062	0.103	0.009	0.022	0.102	0.340	0.000	0.000
	TNF	0.292	0.389	0.003	0.012	0.733	0.733	0.000	0.000
LPS	IL-1β	0.153	0.612	0.119	0.238	0.963	0.963	0.067	0.202
	IL-6	0.368	0.655	0.674	0.674	0.857	0.956	0.182	0.182
	IL-10	0.002	0.005	0.012	0.024	0.317	0.528	0.001	0.005
	IL-23	0.002	0.008	0.025	0.050	0.039	0.117	0.003	0.009
	TNF	0.017	0.068	0.039	0.078	0.498	0.733	0.008	0.016
LTA	IL-1β	0.674	0.878	0.442	0.589	0.593	0.963	0.428	0.428
	IL-6	0.655	0.655	0.563	0.674	0.150	0.482	0.142	0.182
	IL-10	0.378	0.420	0.203	0.254	0.754	0.898	0.004	0.010
	TNF	0.869	0.869	0.829	0.829	0.668	0.733	0.015	0.020
OVA	CCL17	0.329	0.494	0.370	0.370	0.012	0.069	0.062	0.162
	CXCL10	0.381	0.381	0.923	0.923	0.295	0.442	0.160	0.240
	IFN-γ	0.003	0.009	0.004	0.008	0.048	0.144	0.001	0.002
	IL-4	0.198	0.326	0.071	0.095	0.052	0.104	0.023	0.046
	IL-5	0.226	0.271	0.137	0.164	0.188	0.310	0.175	0.210
	IL-10	0.235	0.294	0.760	0.760	0.486	0.694	0.211	0.234
	IL-13	0.294	0.353	0.052	0.078	0.104	0.208	0.088	0.132
	IL-17A	0.352	0.477	0.019	0.028	0.640	0.962	0.054	0.108
Tetanus	CCL17	0.079	0.237	0.021	0.042	0.023	0.069	0.045	0.162
	CXCL10	0.092	0.184	0.134	0.402	0.283	0.442	0.281	0.337
	IFN-γ	0.038	0.057	0.000	0.000	0.001	0.006	0.001	0.002
	IL-4	0.000	0.001	0.000	0.000	0.002	0.008	0.000	0.000
	IL-5	0.118	0.226	0.000	0.000	0.001	0.003	0.001	0.003
	IL-10	0.001	0.005	0.004	0.013	0.185	0.370	0.025	0.036
	IL-13	0.100	0.150	0.000	0.000	0.004	0.024	0.036	0.108
	IL-17A	0.184	0.368	0.015	0.028	0.216	0.648	0.094	0.138
